# Clinical and Microbiological Profile of Culture-Positive Neonatal Sepsis in a Tertiary Care Center in North India

**DOI:** 10.7759/cureus.92136

**Published:** 2025-09-12

**Authors:** Kartik Kartik, Faraz A Khan, Ekansh Rathoria, Divakar Srivastava, Saurabh K Singh, Richa Rathoria

**Affiliations:** 1 Pediatrics, Hind Institute of Medical Sciences, Sitapur, IND; 2 Pediatrics, Autonomous State Medical College, Amethi, IND; 3 Microbiology, Hind Institute of Medical Sciences, Sitapur, IND; 4 Obstetrics and Gynecology, Hind Institute of Medical Sciences, Sitapur, IND

**Keywords:** antibacterial agents, antibiotic resistance, antimicrobial stewardship, blood culture, india, multidrug-resistant organisms, neonatal intensive care units, neonatal sepsis, resource-limited settings, risk factors

## Abstract

Background

Sepsis in newborns is a major clinical concern, especially in resource-limited settings. Although blood culture remains the primary method for confirming infection and guiding treatment, its detection rate is often suboptimal. The objective of this study was to evaluate the incidence, clinical characteristics, maternal and neonatal risk factors, and antimicrobial resistance patterns linked to culture-confirmed neonatal sepsis in a tertiary care facility in North India.

Materials and methods

This prospective observational study was carried out over 18 months in the neonatal intensive care unit (NICU) of Hind Institute of Medical Sciences, a tertiary care teaching hospital in Sitapur, India, enrolling 118 neonates aged 28 days or younger who presented with clinical signs suggestive of sepsis. Data on maternal and neonatal risk factors were collected using a pre-tested proforma. Blood cultures were processed using standard microbiological techniques, and antibiotic susceptibility was assessed. Statistical analysis was performed using IBM SPSS Statistics version 26.0. Significant risk factors associated with culture-positive sepsis were identified using binary logistic regression analysis.

Results

Among 118 neonates, 28% (33/118) had culture-positive sepsis. *Klebsiella pneumoniae* (13/33, 39.4%), *Staphylococcus aureus* (9/33, 27.3%), and *Escherichia coli* (6/33, 18.2%) were the predominant isolates. Most pathogens exhibited resistance to ampicillin and third-generation cephalosporins, while retaining sensitivity to meropenem and amikacin. Significant neonatal risk factors included low birth weight (<2500 g; odds ratio (OR) = 7.179; p < 0.001), resuscitation at birth (OR = 4.222; p = 0.005), positive sepsis screen (OR = 12.03; p < 0.001), and prolonged hospital stay (>7 days; OR = 20.844; p < 0.001). Significant maternal predictors included preterm delivery (OR = 15.80; p < 0.001), premature rupture of membranes ≥18 hours (OR = 4.80; p < 0.001), maternal fever (OR = 5.213; p < 0.001), foul-smelling liquor (OR = 4.875; p = 0.001), ≥3 per vaginal examinations (OR = 4.168; p = 0.001), and cesarean delivery (OR = 3.503; p = 0.004). The sepsis screening test demonstrated a sensitivity of 84.8% and a negative predictive value of 92.1%.

Conclusion

Neonatal sepsis continues to impose a significant clinical burden, with high rates of early-onset sepsis and multidrug-resistant gram-negative organisms. Identification of key maternal and neonatal risk factors may aid early diagnosis and improve outcomes. Strengthening microbiological surveillance and antimicrobial stewardship is essential to combat rising resistance and reduce neonatal mortality.

## Introduction

The neonatal period, the first 28 days of life, is a critical window during which newborns undergo profound physiological adjustments [1]. However, this transition from intrauterine to extrauterine life is fraught with vulnerability, particularly due to an underdeveloped immune system that lacks adequate humoral, cellular, and phagocytic function as well as complement activity, predisposing neonates to serious infections [2].

Neonatal sepsis is defined as a clinical condition presenting with symptoms and signs of infection in a newborn, confirmed by the presence of a pathogen in blood culture or other microbiological tests [3]. About 22% of neonatal deaths worldwide each year are caused by neonatal sepsis, which continues to be one of the leading causes of sickness and death in infants [4]. In high- and middle-income nations, the estimated incidence is approximately 2,200 cases per 100,000 live births, translating to nearly three million cases annually, with mortality ranging from 11% to 19% [4]. Neonates in low- and middle-income countries (LMICs) face significantly higher risks of sepsis, up to 40 times greater than in high-income countries, due to a high burden of infections and inadequate access to quality healthcare [4,5].

India reports the highest incidence of clinical neonatal sepsis at 17,000 cases per 100,000 live births, with a case fatality rate ranging from 25% to 65% [6]. According to the National Family Health Survey (NFHS-5), India’s neonatal mortality rate is 24.9 per 1,000 live births, with prematurity, sepsis, and birth asphyxia being the leading causes of these deaths [7]. Sub-target 3.2 of the Sustainable Development Goals (SDGs), which were introduced in 2016, calls for all countries to reduce neonatal mortality to less than 12 per 1,000 live births and under-five mortality to less than 25 per 1,000 live births by 2030 in order to eliminate preventable deaths among newborns and children under five [8]. Achieving this target necessitates early diagnosis, rational antimicrobial use, and identification of modifiable risk factors, particularly in resource-limited settings.

Neonatal sepsis is commonly defined as early-onset sepsis (EOS), occurring during the first 72 hours after birth, and late-onset sepsis (LOS), which arises beyond the initial 72 hours of life [3]. EOS is predominantly caused by organisms acquired from the maternal genital tract, such as Group B *Streptococcus *(GBS) and *Escherichia coli* *(E. coli)*, while LOS is often hospital-acquired and attributed to organisms like *Staphylococcus aureus*, *Klebsiella pneumoniae*, and *Candida *species [4,9].

Compounding this challenge is the alarming rise of multidrug-resistant (MDR) pathogens, including extended-spectrum beta-lactamase (ESBL) producing* E. coli*, carbapenem-resistant enterobacterales (CRE), and methicillin-resistant *Staphylococcus aureus* (MRSA) [10]. The indiscriminate and empirical use of antibiotics, often without culture sensitivity guidance, has contributed significantly to this resistance crisis [10].

Perinatal risk factors for neonatal sepsis include birth asphyxia, meconium-stained amniotic fluid, maternal colonization with GBS, chorioamnionitis, prolonged rupture of membranes (PROM) >=18 hours, preterm birth (<37 weeks gestational age), maternal urinary or genital tract infections, intrapartum fever, very low birth weight, and three or more vaginal examinations during labor [11].

Despite advances in neonatal care, the diagnosis of sepsis remains a challenge due to nonspecific clinical manifestations and limitations of diagnostic tests [12]. This underscores the need for region-specific studies, such as ours, to evaluate clinical predictors and microbiological profiles that may help guide early diagnosis. Blood culture remains the gold standard for confirmation, but is hindered by low sensitivity and time constraints [12]. As a result, clinicians often rely on sepsis screening tests and initiate empirical antibiotics, which may lead to inappropriate use and the emergence of MDR pathogens [12].

Given the high burden of neonatal sepsis, this study was undertaken to assess the incidence, clinical profile, and outcomes of culture-proven neonatal sepsis in a tertiary care center in North India. The study objectives were to determine the proportion of neonates with blood culture-positive sepsis among suspected cases, examine associated maternal and neonatal risk factors, and analyze the antibiotic susceptibility profiles of the causative organisms.

## Materials and methods

This hospital-based observational study was conducted in the neonatal intensive care unit (NICU) of Hind Institute of Medical Sciences, a tertiary care teaching hospital in North India over 18 months, from August 2023 to January 2025. The study was approved by the Institutional Ethics Committee of Hind Institute of Medical Sciences, Sitapur (no. IHEC-HIMSA/MD-MS-22/RD-30/07-23, dated 12-07-2023), and written informed consent was obtained from the parents or legal guardians of all enrolled neonates. The study included neonates aged ≤28 days who were admitted with clinical suspicion of sepsis and underwent blood culture testing as part of their diagnostic work-up.

Neonates aged ≤28 days admitted to the NICU or neonatal ward with clinical features suggestive of sepsis, such as fever or hypothermia, lethargy, poor feeding, respiratory distress, apnea, seizures, jaundice within the first 24 hours, signs of shock, or abdominal distension, were included. Only those for whom blood cultures were obtained before initiation of antibiotics or within 48 hours of therapy were considered. Exclusion criteria included neonates with major congenital anomalies (e.g., cyanotic heart disease, neural tube defects, and chromosomal abnormalities), those already on antibiotic therapy for more than 48 hours before admission, incomplete medical or laboratory records, discharge or death before culture results were available, and those with known inborn errors of metabolism or genetic syndromes potentially confounding the diagnosis of sepsis.

The sample size was calculated using Open-Epi (Open-Source Epidemiologic Statistics for Public Health) software, an open-access online tool. Based on an 8.3% incidence of culture-positive neonatal sepsis reported in a previous study, with a fixed population size of 1500, a 95% confidence level, and a 5% margin of error, the minimum required sample size was 109 [13,14]. However, a total of 118 neonates were enrolled using consecutive sampling to enhance statistical power and account for any potential exclusions or missing data.

Data were collected using a structured, pre-tested proforma capturing maternal and neonatal demographics, clinical features, and outcomes. Maternal variables included age (<=25 and >25 years), number of antenatal visits (<8 or ≥8), presence of foul-smelling liquor, fever, urinary tract infection (UTI), ≥3 per vaginal (PV) examinations, PROM ≥18 hours, gestational age (<37 weeks as preterm or ≥37 weeks as term), place of delivery (inborn or out-born), and mode of delivery (vaginal or lower segment caesarean section [LSCS]) [11]. Neonatal variables included age at admission (<72 hours as EOS or ≥72 hours as LOS), gender (male or female), birth weight (<2500 or ≥2500 grams), need for resuscitation at birth, clinical signs (fever, lethargy, poor feeding, respiratory distress, seizures), meconium-stained liquor (MSL), duration of hospital stay (≤7 or >7 days), outcome, sepsis screen results, and blood culture findings with antibiotic sensitivity and resistance patterns. The maternal age cutoff of 25 years was chosen post hoc, as it provided balanced groups in our cohort.

A positive sepsis screen was defined as the presence of two or more abnormal parameters among the following: C-reactive protein (CRP) >12 mg/dL, absolute neutrophil count (ANC) <1500/mm³, total leukocyte count (TLC) <5000/mm³, erythrocyte sedimentation rate (ESR) >15 mm in the first hour, and an immature-to-total neutrophil (I/T) ratio >0.2 [15].

Under aseptic conditions, 1-2 mL of venous blood was collected and inoculated into brain heart infusion (BHI) broth, then incubated and sub-cultured on blood and MacConkey agar. Bacterial identification was carried out using standard biochemical methods, and antibiotic susceptibility testing was performed using the Kirby-Bauer disc diffusion method, interpreted according to Clinical and Laboratory Standards Institute (CLSI) breakpoints [16]. Antibiotic susceptibility testing followed standard laboratory protocols for neonatal isolates; selected organism-drug combinations were not tested when agents were clinically inapplicable, organism-/age-specific breakpoints were unavailable, or testing was excluded from the routine neonatal panel because of sample-volume or resource limitations. No molecular techniques such as PCR (polymerase chain reaction), MALDI-TOF (matrix-assisted laser desorption/ionization-time-of-flight mass spectrometry), or next-generation sequencing were used because of financial and resource limitations.

Culture-positive sepsis was defined as the isolation of a pathogenic organism from blood in neonates with clinical signs of sepsis [16]. MDR organisms were those resistant to at least one agent in three or more antimicrobial classes [17]. The primary outcome was culture-confirmed neonatal sepsis, with analysis of bacterial isolates and their antibiotic susceptibility profiles. Secondary outcomes included associations between culture positivity and maternal, neonatal, and clinical variables, as well as indicators of neonatal morbidity such as hospital stay duration and sepsis-related complications.

Data were entered and analyzed using IBM SPSS Statistics for Windows, version 26.0 (IBM Corp., Armonk, NY, USA). Categorical variables were expressed as frequencies and percentages, while continuous variables were summarized using means and standard deviations. The Chi-square test was used to assess associations between categorical variables. Binary logistic regression analysis was performed to identify maternal and neonatal risk factors independently associated with culture-proven neonatal sepsis. Odds ratios (ORs) with 95% confidence intervals (CIs) were reported. A p-value of <0.05 was considered statistically significant.

## Results

This study included 118 neonates. The mean age of neonates at admission was 18.2 ± 27.6 hours, and the mean duration of hospital stay was 8.76 ± 4.26 days. The majority (99/118, 83.9%) had EOS (<72 hours) (Table 1). Most were male (70/118, 59.3%) and had a birth weight ≥2500 grams (83/118, 70.3%). Common presenting features included respiratory distress (67/118, 56.8%) and poor feeding (22/118, 18.6%). Sepsis screening was positive in 46.6% (55/118). The mortality rate was 8.5% (10/118). A total of 33/118 (28%) neonates were culture-positive, while 85/118 (72%) were culture-negative. Culture positivity was significantly associated with low birth weight (p < 0.0001), need for resuscitation at birth (p = 0.003), prolonged hospital stays (p < 0.0001), and positive sepsis screening (p < 0.0001), all of which may help in early identification of neonates at increased risk for bloodstream infections.

**Table 1:** Association between demographic and clinical parameters of neonates with neonatal blood culture positivity (n = 118)* *MSL: meconium-stained liquor, χ²: Chi-square test statistic, r: point-biserial correlation coefficient, p (r): p-value associated with r, data presented as n (%), significant p-value <0.05.

Characteristics		Total (n = 118)	Culture negative (n = 85)	Culture positive (n = 33)	χ^2^	p-value	r	p (r)
Age at admission (hours)	<72	99 (83.9)	74 (87.1)	25 (75.8)	2.247	0.134	0.138	0.136
	≥72	19 (16.1)	11 (12.9)	8 (24.2)				
Gender	Female	48 (40.7)	33 (38.8)	15 (45.5)	0.433	0.510	-0.061	0.515
	Male	70 (59.3)	52 (61.2)	18 (54.5)				
Birth weight (grams)	<2500	35 (29.7)	15 (17.6)	20 (60.6)	21.027	<0.0001	-0.422	<0.0001
	≥2500	83 (70.3)	70 (82.4)	13 (39.4)				
Resuscitation at birth required	No	98 (83.1)	76 (89.4)	22 (66.7)	8.736	0.003	0.272	0.003
	Yes	20 (16.9)	9 (10.6)	11 (33.3)				
Fever	No	107 (90.7)	79 (92.9)	28 (84.8)	1.842	0.175	0.125	0.178
	Yes	11 (9.3)	6 (7.1)	5 (15.2)				
Lethargy	No	102 (86.4)	74 (87.1)	28 (84.8)	0.099	0.753	0.029	0.755
	Yes	16 (13.6)	11 (12.9)	5 (15.2)				
Poor feeding	No	96 (81.4)	69 (81.2)	27 (81.8)	0.006	0.936	-0.007	0.937
	Yes	22 (18.6)	16 (18.8)	6 (18.2)				
Respiratory distress	No	51 (43.2)	36 (42.4)	15 (45.5)	0.093	0.760	-0.028	0.763
	Yes	67 (56.8)	49 (57.6)	18 (54.5)				
MSL	No	98 (83.1)	72 (84.7)	26 (78.8)	0.591	0.442	0.071	0.446
	Yes	20 (16.9)	13 (15.3)	7 (21.2)				
Neonatal seizure	No	108 (91.5)	80 (94.1)	28 (84.8)	2.633	0.105	0.149	0.106
	Yes	10 (8.5)	5 (5.9)	5 (15.2)				
Duration of hospital stay (days)	<=7	72 (61.0)	72 (84.7)	0 (0)	40.516	<0.0001	0.586	<0.0001
	>7	46 (39.0)	13 (15.3)	33 (100)				
Outcome	Death	10 (8.5)	5 (5.9)	5 (15.2)	2.633	0.105	-0.149	0.106
	Discharge	108 (91.5)	80 (94.1)	28 (84.8)				
Sepsis screening	Negative	63 (53.4)	58 (68.2)	5 (15.2)	26.918	<0.0001	0.478	<0.0001
	Positive	55 (46.6)	27 (31.8)	28 (84.8)				

The mean maternal age was 28.8 ± 7 years. Most mothers were over 25 years of age (77/118, 65.3%) and had fewer than eight antenatal visits (107/118, 90.7%) (Table 2). Common risk factors included maternal fever (29/118, 24.6%), PROM (47/118, 39.8%), and ≥3 PV examinations (37/118, 31.4%). A majority of deliveries occurred at term (94/118, 79.7%) and within the hospital (82/118, 69.5%), with vaginal delivery being more frequent (84/118, 71.2%). Significant predictors included foul-smelling liquor, maternal fever, ≥3 vaginal examinations, PROM, preterm gestation, and caesarean delivery, all of which were significantly associated with higher rates of culture-positive neonatal sepsis (p < 0.05).

**Table 2:** Association between demographic and clinical parameters of mothers with neonatal blood culture positivity (n = 118)* *GA: gestational age, LSCS: lower segment caesarean section, PROM: premature rupture of membranes, PV: per vaginal, UTI: urinary tract infection, χ²: Chi-square test statistic, r: point-biserial correlation coefficient, p (r): p-value associated with r, data presented as n (%), significant p-value < 0.05.

Characteristics		Total (n = 118)	Culture negative (n = 85)	Culture positive (n = 33)	χ^2^	p-value	r	p (r)
Maternal age (years)	<=25	41 (34.7)	32 (37.6)	9 (27.3)	1.128	0.288	0.098	0.292
	>25	77 (65.3)	53 (62.4)	24 (72.7)				
Antenatal visits	<8	107 (90.7)	79 (92.9)	28 (84.8)	1.842	0.175	0.125	0.178
	>=8	11 (9.3)	6 (7.1)	5 (15.2)				
Foul-smelling liquor	No	95 (80.5)	75 (88.2)	20 (60.6)	11.564	<0.001	0.313	<0.001
	Yes	23 (19.5)	10 (11.8)	13 (39.4)				
Maternal fever	No	89 (75.4)	72 (84.7)	17 (51.5)	14.127	<0.0001	0.346	<0.0001
	Yes	29 (24.6)	13 (15.3)	16 (48.5)				
Maternal UTI	No	102 (86.4)	76 (89.4)	26 (78.8)	2.289	0.130	0.139	0.133
	Yes	16 (13.6)	9 (10.6)	7 (21.2)				
≥3 PV examinations	No	81 (68.6)	66 (77.6)	15 (45.5)	11.446	<0.001	0.311	<0.001
	Yes	37 (31.4)	19 (22.4)	18 (54.5)				
PROM	No	71 (60.2)	60 (70.6)	11 (33.3)	13.767	<0.0001	0.342	<0.0001
	Yes	47 (39.8)	25 (29.4)	22 (66.7)				
GA	Pre-term	24 (20.3)	6 (7.1)	18 (54.5)	33.084	<0.0001	-0.530	<0.0001
	Term	94 (79.7)	79 (92.9)	15 (45.5)				
Place of delivery	Inborn	82 (69.5)	58 (68.2)	24 (72.7)	0.226	0.634	-0.044	0.638
	Outborn	36 (30.5)	27 (31.8)	9 (27.3)				
Mode of delivery	Vaginal	84 (71.2)	67 (78.8)	17 (51.5)	8.643	0.003	0.271	0.003
	LSCS	34 (28.8)	18 (21.2)	16 (48.5)				

**Figure 1:**
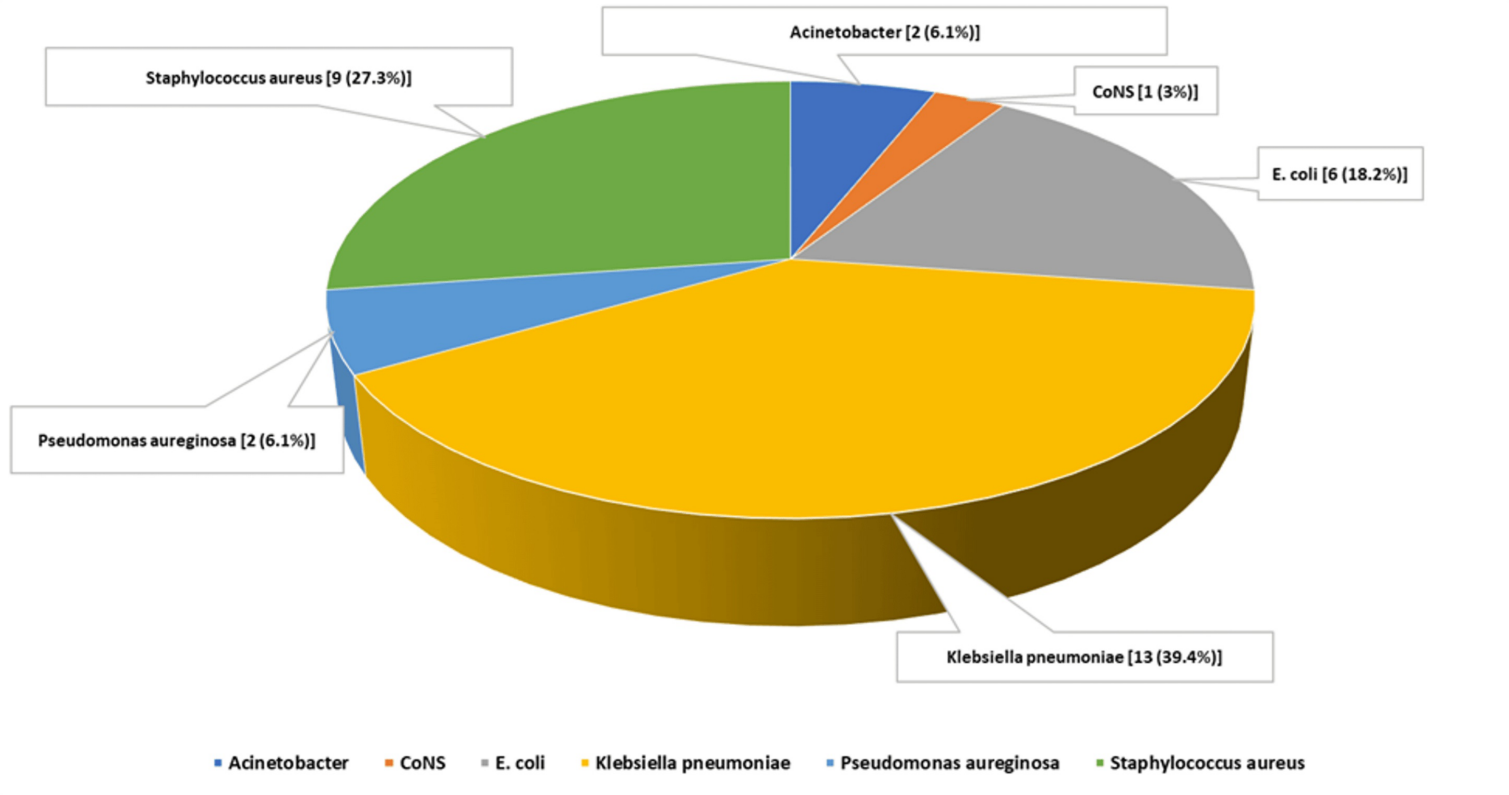
Isolated organisms in blood culture positive neonates (n = 33)* *CoNS: coagulase-negative* Staphylococci*; *E. coli: Escherichia coli*; data presented as n (%); percentages may not total to 100 due to rounding.

*Klebsiella pneumoniae *was the most frequently isolated organism (13/33, 39.4%), followed by *S. aureus* (9/33, 27.3%) and *E. coli* (6/33, 18.2%), highlighting the predominance of gram-negative sepsis in this cohort (Figure 1).

*K. pneumoniae* and *E. coli* exhibited high sensitivity to meropenem (100%) and moderate resistance to third-generation cephalosporins and ampicillin (Table 3). *S. aureus* showed full susceptibility to vancomycin and linezolid, while exhibiting high resistance to ciprofloxacin and ampicillin. These patterns highlight a high prevalence of multidrug-resistant organisms, emphasizing the need for robust antimicrobial stewardship.

**Table 3:** Antibiotic susceptibility patterns of bacterial isolates from neonatal blood cultures (n = 33)* *S: sensitive, R: resistant, CoNS: coagulase-negative* Staphylococci*, *E. coli: Escherichia coli*, *P. aeruginosa: Pseudomonas aeruginosa*, *S. aureus: Staphylococcus* *aureus*, NT: not tested; data presented as n (%).

Antibiotic	*Acinetobacter* (n = 2)		*CoNS* (n = 1)		*E. coli *(n = 6)		*Klebsiella pnuemoniae* (n = 13)		*P. aeruginosa* (n = 2)		*S. aureus *(n = 9)	
	S	R	S	R	S	R	S	R	S	R	S	R
Amikacin	1 (50)	1 (50)	0 (0)	1 (100)	6 (100)	0 (0)	10 (76.9)	3 (23.1)	2 (100)	0 (0)	7 (77.8)	2 (22.2)
Cefotaxime	1 (50)	1 (50)	0 (0)	1 (100)	3 (50)	3 (50)	5 (38.5)	8 (61.5)	1 (50)	1 (50)	6 (66.7)	3 (33.3)
Cefepime	2 (100)	0 (0)	NT	NT	6 (100)	0 (0)	9 (69.2)	4 (30.8)	2 (100)	0 (0)	NT	NT
Ampicillin	0 (0)	2 (100)	0 (0)	1 (100)	0 (0)	6 (100)	0 (0)	13 (100)	0 (0)	2 (100)	1 (11.1)	8 (88.9)
Ciprofloxacin	0 (0)	2 (100)	0 (0)	1 (100)	2 (33.3)	4 (66.7)	4 (30.8)	9 (69.2)	0 (0)	2 (100)	1 (11.1)	8 (88.9)
Piperacillin-tazobactam	1 (50)	1 (50)	NT	NT	NT	NT	4 (30.8)	9 (69.2)	1 (50)	1 (50)	NT	NT
Meropenem	2 (100)	0 (0)	NT	NT	6 (100)	0 (0)	13 (100)	0 (0)	2 (100)	0 (0)	NT	NT
Vancomycin	NT	NT	1 (100)	0 (0)	NT	NT	NT	NT	NT	NT	9 (100)	0 (0)
Linezolid	NT	NT	1 (100)	0 (0)	NT	NT	NT	NT	NT	NT	9 (100)	0 (0)

The sepsis screening test demonstrated a sensitivity of 84.8%, a specificity of 68.2%, a positive predictive value (PPV) of 50.9%, and a negative predictive value (NPV) of 92.1%. The overall diagnostic accuracy was 72.9%, indicating a good ability to rule out sepsis when the screen is negative.

Binary logistic regression revealed that culture-positive neonatal sepsis was significantly associated with premature rupture of membranes (OR 4.80, 95% CI: 2.03-11.36, p < 0.001), maternal fever (OR 5.21, 95% CI: 2.11-12.86, p < 0.001), foul-smelling amniotic fluid (OR 4.88, 95% CI: 1.87-12.74, p = 0.001), and three or more per vaginal examinations (OR 4.17, 95% CI: 1.77-9.80, p = 0.001) (Table 4). Preterm birth was the strongest predictor (OR = 15.80, 95% CI: 5.39-46.35, p < 0.001), followed by caesarean delivery (OR = 3.50, 95% CI: 1.49-8.27, p = 0.004). Other variables such as maternal age, antenatal visit frequency, maternal UTI, and place of birth did not show statistically significant associations.

**Table 4:** Binary logistic regression analysis of maternal risk factors associated with neonatal blood culture positivity (n = 118)* *B: regression coefficient, S.E.: standard error, OR: odds ratio, CI: confidence interval, PROM: premature rupture of membranes, PV: per vaginum, LSCS: lower segment cesarean section, UTI: urinary tract infection; significant p-value <0.05.

Variable	B	S.E.	Wald	p-value	OR [Exp(B)]	95% CI for OR
Maternal age group (≤25 vs >25 years)	0.476	0.450	1.118	0.290	1.610	0.666–3.893
Antenatal visit (<8 vs ≥8)	0.855	0.644	1.761	0.184	2.351	0.665–8.311
Foul-smelling liquor (Yes vs. No)	1.584	0.490	10.445	0.001	4.875	1.865–12.741
Maternal fever (Yes vs. No)	1.651	0.461	12.851	<0.001	5.213	2.114–12.856
Maternal UTI (Yes vs. No)	0.821	0.553	2.207	0.137	2.274	0.769–6.718
≥3 PV examinations (Yes vs. No)	1.428	0.436	10.725	0.001	4.168	1.774–9.795
PROM (Yes vs. No)	1.569	0.439	12.747	<0.001	4.800	2.029–11.356
Mode of delivery (LSCS vs. vaginal)	1.254	0.438	8.195	0.004	3.503	1.485–8.265
Gestational age (preterm vs. term)	2.760	0.549	25.262	<0.001	15.800	5.386–46.353
Place of birth (inborn vs. outborn)	0.216	0.455	0.226	0.635	1.241	0.509–3.029

An analysis using binary logistic regression showed that low birth weight (OR = 7.18, 95% CI: 2.94-17.55, p < 0.001), need for resuscitation at birth (OR = 4.22, 95% CI: 1.55-11.48, p = 0.005), positive sepsis screening (OR = 12.03, 95% CI: 4.19-34.57, p < 0.001), and prolonged duration of hospital stay (OR = 20.84, 95% CI: 7.05-61.66, p < 0.001) were significantly associated with increased odds of culture-positive neonatal sepsis (Table 5). Other factors such as gender, age at admission, fever, lethargy, poor feeding, respiratory distress, presence of meconium-stained liquor, neonatal seizures, and outcome were not statistically significant predictors.

**Table 5:** Binary logistic regression analysis of neonatal risk factors associated with neonatal blood culture positivity (n = 118)* *B: regression coefficient, S.E.: standard error, OR: odds ratio, CI: confidence interval; significant p-value <0.05.

Variable	B	S.E.	Wald	p-value	OR (Exp(B))	95% CI for OR
Age at admission (<72 hours vs. >=72 hours	0.767	0.519	2.182	0.140	2.153	0.778–5.954
Gender (male vs. female)	-0.272	0.414	0.432	0.511	0.762	0.338–1.716
Birth weight (low vs. normal)	1.971	0.456	18.693	<0.001	7.179	2.938–17.546
Resuscitation at birth (Yes vs. No)	1.440	0.511	7.960	0.005	4.222	1.552–11.484
Fever (Yes vs. No)	0.855	0.644	1.761	0.184	2.351	0.665–8.311
Lethargy (Yes vs. No)	0.183	0.583	0.099	0.753	1.201	0.383–3.768
Poor feeding (Yes vs. No)	-0.043	0.530	0.006	0.936	0.958	0.339–2.707
Respiratory distress (Yes vs. No)	-0.126	0.413	0.093	0.760	0.882	0.393–1.980
Meconium-stained liquor (Yes vs. No)	0.400	0.522	0.587	0.444	1.491	0.536–4.145
Neonatal seizure (Yes vs. No)	1.050	0.669	2.459	0.117	2.857	0.769–10.612
Sepsis screening positive (Yes vs. No)	2.487	0.539	21.335	<0.001	12.030	4.187–34.565
Duration of hospital stay (<=7 days vs. >7 days)	3.037	0.553	30.124	<0.001	20.844	7.047–61.660
Outcome (discharged vs. death)	-1.050	0.669	2.459	0.117	0.350	0.094–1.300

## Discussion

This hospital-based observational study found that 28% (33/118) of neonates with clinical suspicion of sepsis had culture-proven infections, consistent with findings from similar studies in India and other LMICs. This reflects the diagnostic challenge of neonatal sepsis, as the majority of clinically suspected cases were culture-negative, underscoring the limited sensitivity of blood cultures and the need for adjunctive screening tests. For instance, a study by Mohakud et al. in Odisha, India, reported a blood culture positivity rate of 25.7% in neonates, while Bech et al. observed a pooled rate of 26.2% across studies conducted in sub-Saharan Africa [8,16]. Nevertheless, blood culture remains the gold standard for confirming neonatal sepsis, particularly in low-resource settings.

Our study reaffirmed the predominance of EOS (99/118, 83.9%), aligning with Murthy et al.'s meta-analysis, which noted EOS to be more common in LMICs due to perinatal risk factors such as PROM, maternal infections, and unhygienic delivery conditions [6]. The high rate of EOS underscores the importance of intrapartum infection control practices and timely screening of maternal risk factors.

The most frequently isolated organisms were *K. pneumoniae, S. aureus,* and *E. coli,* reflecting the pattern seen in previous Indian studies and WHO surveillance data [4,9]. Wen et al. found the *Klebsiella *species to be the leading pathogen in gram-negative neonatal sepsis in LMICs, often associated with high antimicrobial resistance [4]. Similarly, Zelellw et al. identified *Klebsiella *and *E. coli* as the predominant pathogens associated with neonatal sepsis in their analysis of studies from developing nations [9]. These findings emphasize the importance of tailoring empirical antibiotic regimens to local microbial profiles. Gram-negative organisms were predominant and exhibited significant resistance to first-line antibiotics like ampicillin and cefotaxime, while retaining sensitivity to meropenem and amikacin. These findings are consistent with global data showing rising resistance to beta-lactams and increasing reliance on carbapenems and glycopeptides [3,5].

Importantly, we observed a high prevalence of MDR organisms, particularly among *Klebsiella *and *E. coli* isolates, which showed high resistance to ampicillin and third-generation cephalosporins, but retained good sensitivity to meropenem and amikacin. This trend echoes findings from Wattal et al., who reported MDR rates exceeding 70% in neonatal intensive care settings in India [10]. The rise of MDR pathogens calls for improved antibiotic stewardship, culture-guided therapy, and hospital infection control protocols. Lodhi et al. concluded that the rising prevalence of ESBL and MDR *Klebsiella pneumoniae* in hospital-acquired infections poses a serious public health threat in India, highlighting the need for urgent control through a multidisciplinary One Health approach [18].

A key contribution of our study is the analysis of antimicrobial susceptibility patterns in culture-positive neonates, providing updated local data to guide empirical therapy. The high prevalence of MDR organisms highlights the urgent need for surveillance and standardized definitions to strengthen infection control in neonatal units [17].

Among maternal risk factors, PROM ≥18 hours (OR = 4.80; p < 0.001), maternal fever (OR = 5.213; p <0.001), foul-smelling liquor (OR = 4.875; p = 0.001), and ≥3 per vaginal examinations (OR = 4.168; p = 0.001) were significantly associated with culture-positive sepsis. These findings are in agreement with those of Guo et al., who in a meta-analysis identified PROM, maternal infection, and repeated vaginal examinations as significant predictors of early-onset neonatal sepsis [11]. Andini et al. reported a significantly higher incidence of neonatal sepsis among infants born to mothers with PROM (OR 2.69, 95% CI: 1.56-4.65; p < 0.00001) and among preterm neonates compared to those born at term (OR 2.55, 95% CI: 1.61-4.04; p < 0.00001), findings that align with the results of our study, where gestational age and mode of delivery were considered as separate, independent variables [19]. Bayih et al. reported that neonates born to mothers with antenatal urinary tract infections or intrapartum fever had a significantly increased risk, 3.55 and 3.63 times, respectively, of developing neonatal sepsis compared to those born to mothers without these conditions [20]. Traoré et al. also reported similar findings [21].

In our study, cesarean delivery was independently associated with higher odds of adverse neonatal outcomes (OR = 3.50, 95% CI: 1.49-8.27, p = 0.004), distinct from the effect of prematurity. This association is more likely attributable to the presence of obstetric or fetal complications such as fetal distress, thick meconium-stained liquor, prolonged rupture of membranes, or maternal infections, which often necessitate operative intervention, rather than the surgical mode of delivery itself being a direct causal factor [8,11,22].

In addition, preterm birth emerged as the most significant risk factor in our study (OR = 15.80, p < 0.001), further corroborating the role of gestational maturity in neonatal vulnerability to infection. Similar associations were documented by Guo et al., who identified low birth weight and prematurity as key predictors of neonatal sepsis in their meta-analysis [11].

On the neonatal side, low birth weight (OR = 7.179; p < 0.001), need for resuscitation at birth (OR = 4.222; p = 0.005), positive sepsis screening (OR = 12.03; p < 0.001), and longer hospital stay (>7 days; OR = 20.844; p < 0.001) were independently associated with culture positivity. These associations are well supported by existing literature. For instance, Fleischmann et al. reported that neonates with low birth weight and extended hospitalization had significantly higher sepsis-related mortality and morbidity in their global analysis [5]. Similarly, Adhisivam emphasized the prognostic value of clinical scoring and screening markers in predicting sepsis outcomes in sick neonates [7]. Fu et al. identified neonatal sepsis as a significant risk factor contributing to prolonged NICU stay, highlighting its impact on healthcare burden and neonatal outcomes [23].

The sepsis screening panel used in our study showed a sensitivity of 84.8% and NPV of 92.1%, supporting its role in ruling out sepsis when negative. This aligns with prior reports from Celik et al., who advocated for combining clinical suspicion with a standardized screen to improve diagnostic accuracy [12]. Likewise, Jatsho et al. reported that, using blood culture as the gold standard, a positive sepsis screen demonstrated a sensitivity of 81.8%, specificity of 22.5%, PPV of 15.6%, and NPV of 87.7% for detecting neonatal sepsis [24].

The Every Newborn Action Plan (ENAP), launched in 2014, sets strategic goals to end preventable newborn deaths and stillbirths while also reducing maternal mortality. In 2022, the joint Every Newborn Action Plan - Ending Preventable Maternal Mortality (ENAP-EPMM) Measurement Improvement Roadmap (2023-2025) was introduced to enhance tracking of progress toward these targets [25]. The India Newborn Action Plan (INAP), launched in 2014, aims to eliminate preventable newborn deaths by scaling up proven, cost-effective interventions; its success requires sustained implementation, regular monitoring, and integration into broader maternal and child health programs [26].

The strengths of this study include its prospective design, comprehensive inclusion of both maternal and neonatal variables, and correlation of clinical data with microbiological profiles. The use of standardized diagnostic criteria and robust statistical analysis adds to its credibility.

However, the study has certain limitations. First, being single-centered, the findings may not be generalizable across diverse regions. Second, the relatively modest sample size may limit the statistical power and external validity of the results. Third, the yield of blood cultures may have been influenced by prior antibiotic exposure or low-volume sampling, which can underestimate true sepsis rates. Fourth, viral and fungal causes were not investigated, potentially missing non-bacterial etiologies. Finally, advanced molecular diagnostic techniques such as PCR-based methods, MALDI-TOF, or next-generation sequencing were not employed due to financial and resource constraints, which may have restricted the precise identification of fastidious or atypical organisms. Nonetheless, the conventional microbiological methods used in this study remain standard practice in many tertiary care centers across India. Future multicentric studies with larger sample sizes and incorporation of molecular diagnostic approaches are warranted to validate and extend our findings.

## Conclusions

This study demonstrates that neonatal sepsis remains a major concern in a tertiary care center in North India, with a predominance of early-onset cases. *K. pneumoniae* and *E. coli* were the leading pathogens, with a high proportion exhibiting multidrug resistance. Prematurity, low birth weight, PROM, maternal fever, intrapartum infections, and the need for neonatal resuscitation were identified as significant independent risk factors. Cesarean delivery was also associated with higher odds of neonatal sepsis, most likely reflecting underlying obstetric or fetal complications necessitating operative intervention rather than the surgical mode of delivery itself. These findings emphasize the importance of targeted risk factor identification, timely initiation of appropriate therapy guided by local antimicrobial sensitivity, and strengthening infection control and stewardship programs to improve neonatal outcomes.
